# Supplementary figures and images for: Mobile gene silencing in Arabidopsis is regulated by hydrogen peroxide
Source: PeerJ. 2014 Dec 23;2:e701. doi: 10.7717/peerj.701 (PMC4277490; doi:10.7717/peerj.701)

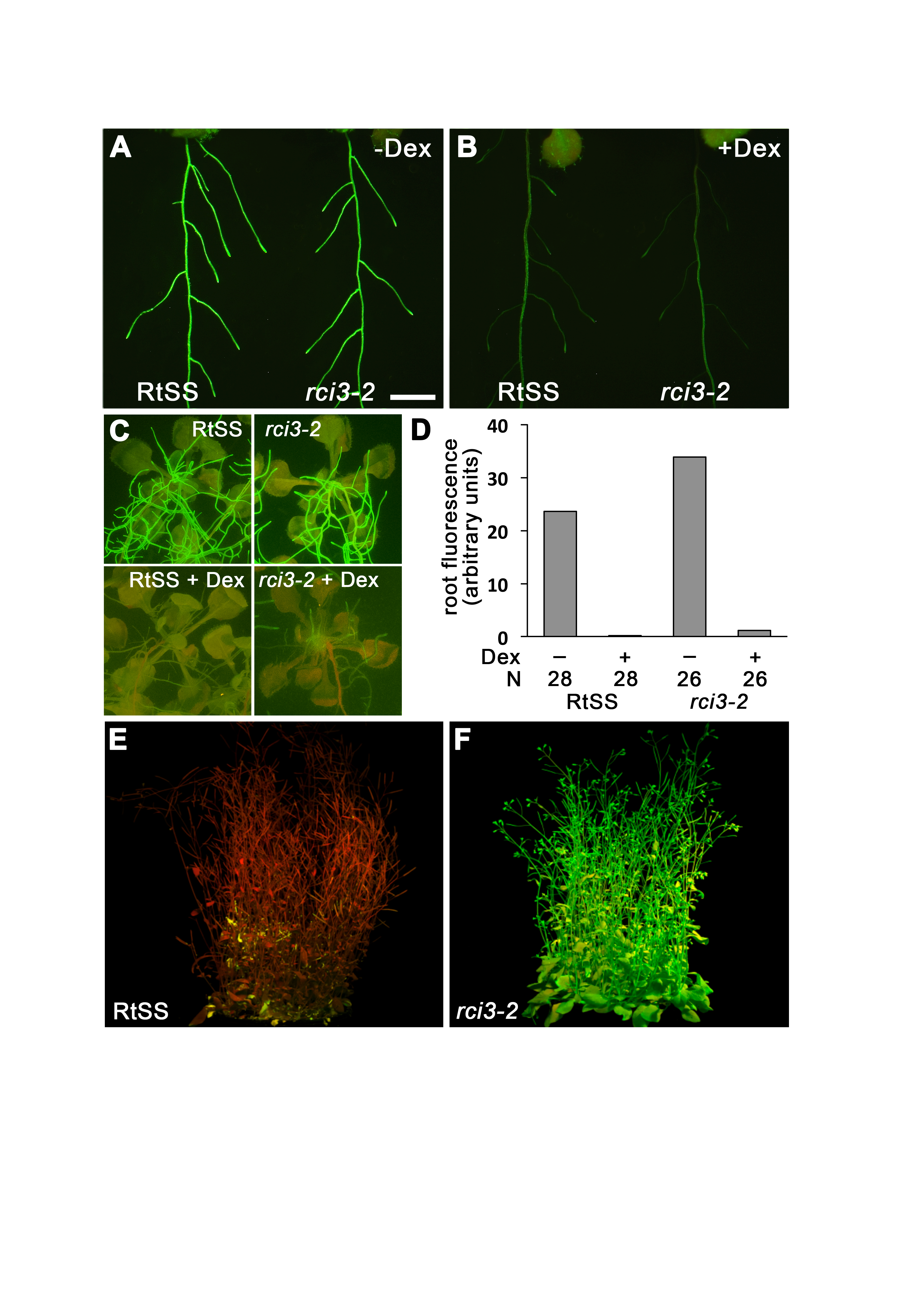

Supplement: Figure S2 — (A) 10-day-old RtSS and mutant line grown without Dex. (B) 10-day-old RtSS and mutant line germinated on medium containing Dex. Bar = 200 µm for (A), (B). (C) RtSS and mutant plants grown without (above) or with (below) Dex for 20 days. (D) Quantification of GFP fluorescence in Dex-induced roots relative to uninduced roots in RtSS and mutant plants. Fluorescence expressed in arbitrary units after subtraction of background fluorescence from GFP fluorescence. N, number of plants. (E) 46-day old RtSS lines with Dex showed complete silencing. (F) 46-day old RtSS lines with Dex showed no silencing in the shoots. [file peerj-02-701-s002.png]

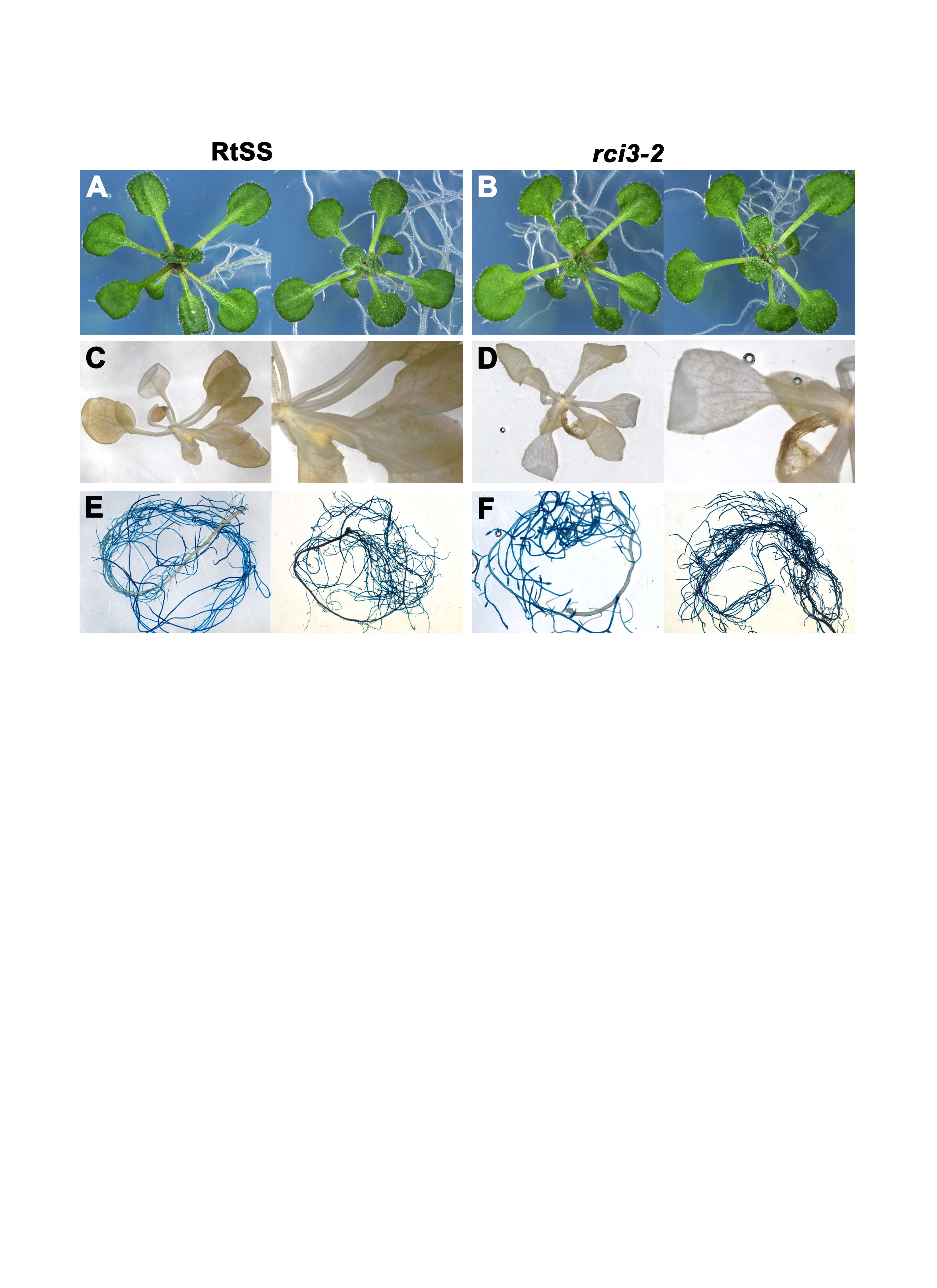

Supplement: Figure S3 — (A) WT RtSS plants. (B) rci3-2 mutants. (C) GUS expression in RtSS shoots, higher magnification at right. (D) GUS expression in rci3-2 shoots, higher magnification at right. (E) GUS expression in RtSS roots. (F) GUS expression in rci3-2 roots. (C–F) Representative images from 12 (RtSS) and 15 (rci3-2) plants grown on Dex then stained with x-gluc substrate for 3 h before imaging. Roots were separated from shoots before x-gluc staining to avoid bleeding of the product from strongly-expressing roots. [file peerj-02-701-s003.png]

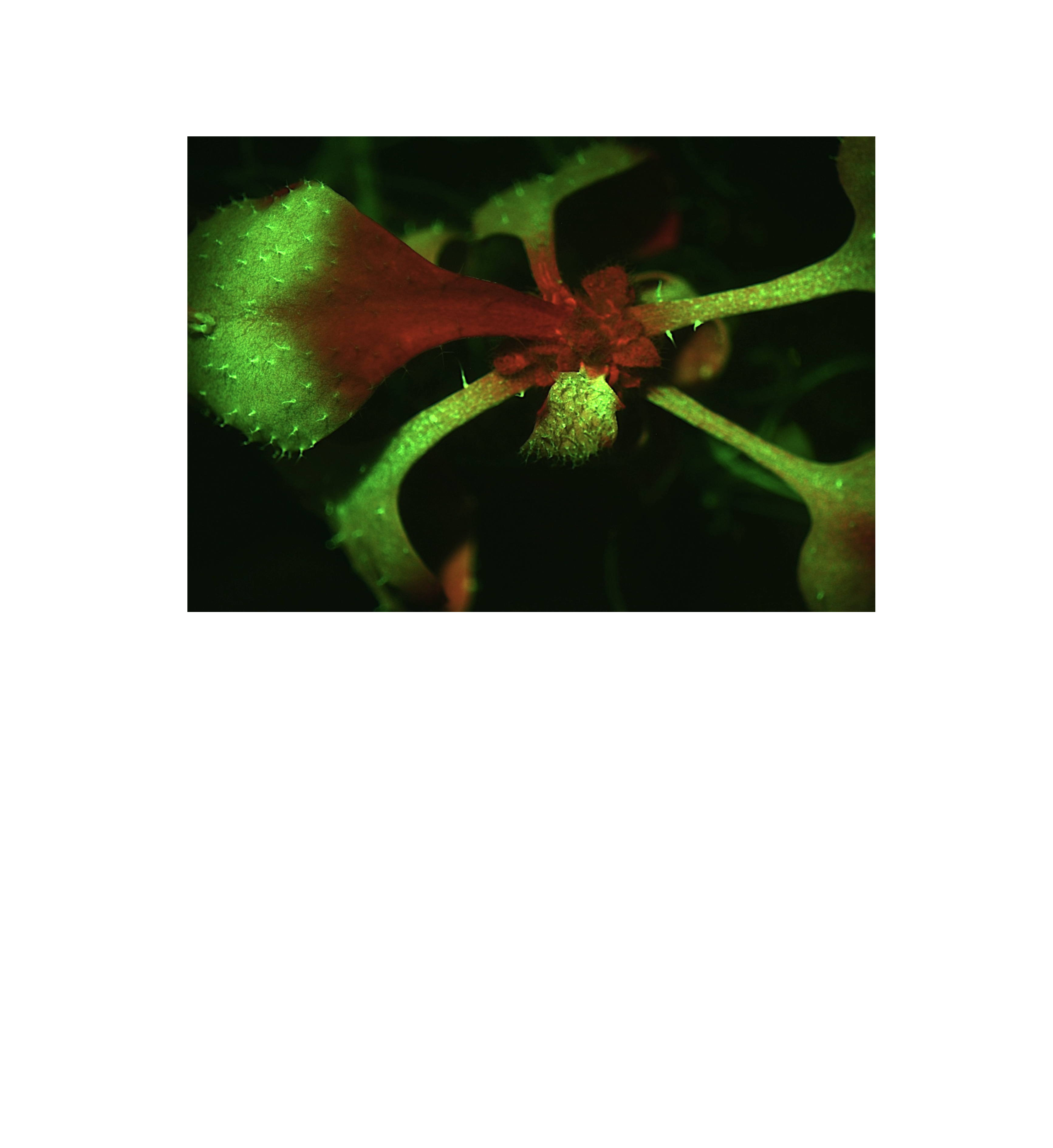

Supplement: Figure S4 — rci3-2 plant, transformed with the 4.5 kb genomic fragment containing RCI3, germinated and grown for 18 days on kanamycin selection medium containing Dex. GFP silencing has spread from the root into the youngest shoot tissue. [file peerj-02-701-s004.png]

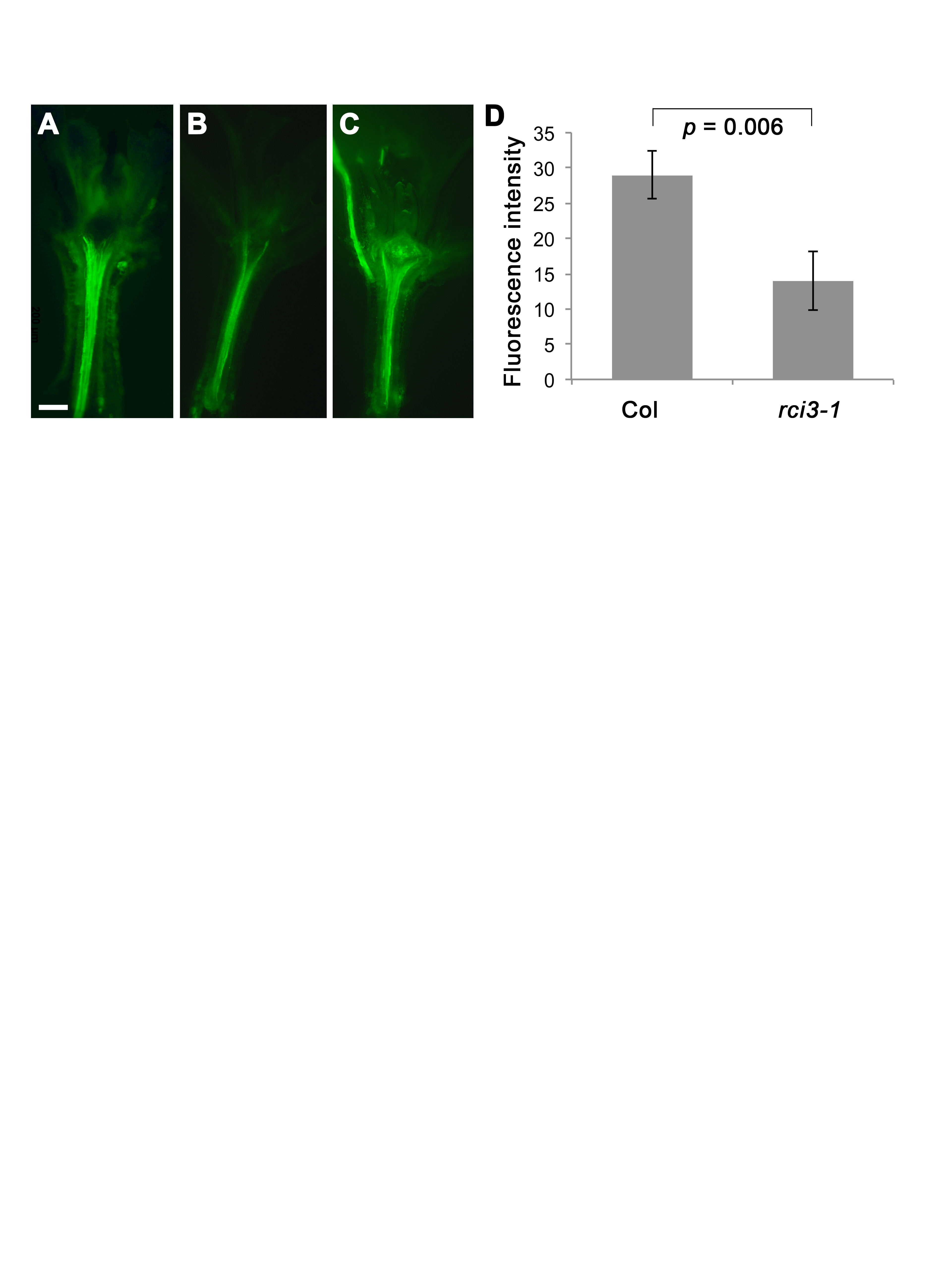

Supplement: Figure S5 — (A) WT Col plant (B) rci3-2011;1 T-DNA insertion line (C) rci3-1 line treated with 1.5 mM H2O2 before staining (D) Fluorescence intensity (arbitrary units) in the hypocotyl stele of Col (n = 19) and rci3-1 (n = 14) plants. Mean ± standard error of the mean shown. P value from two-tailed unpaired Student’s t test [file peerj-02-701-s005.png]

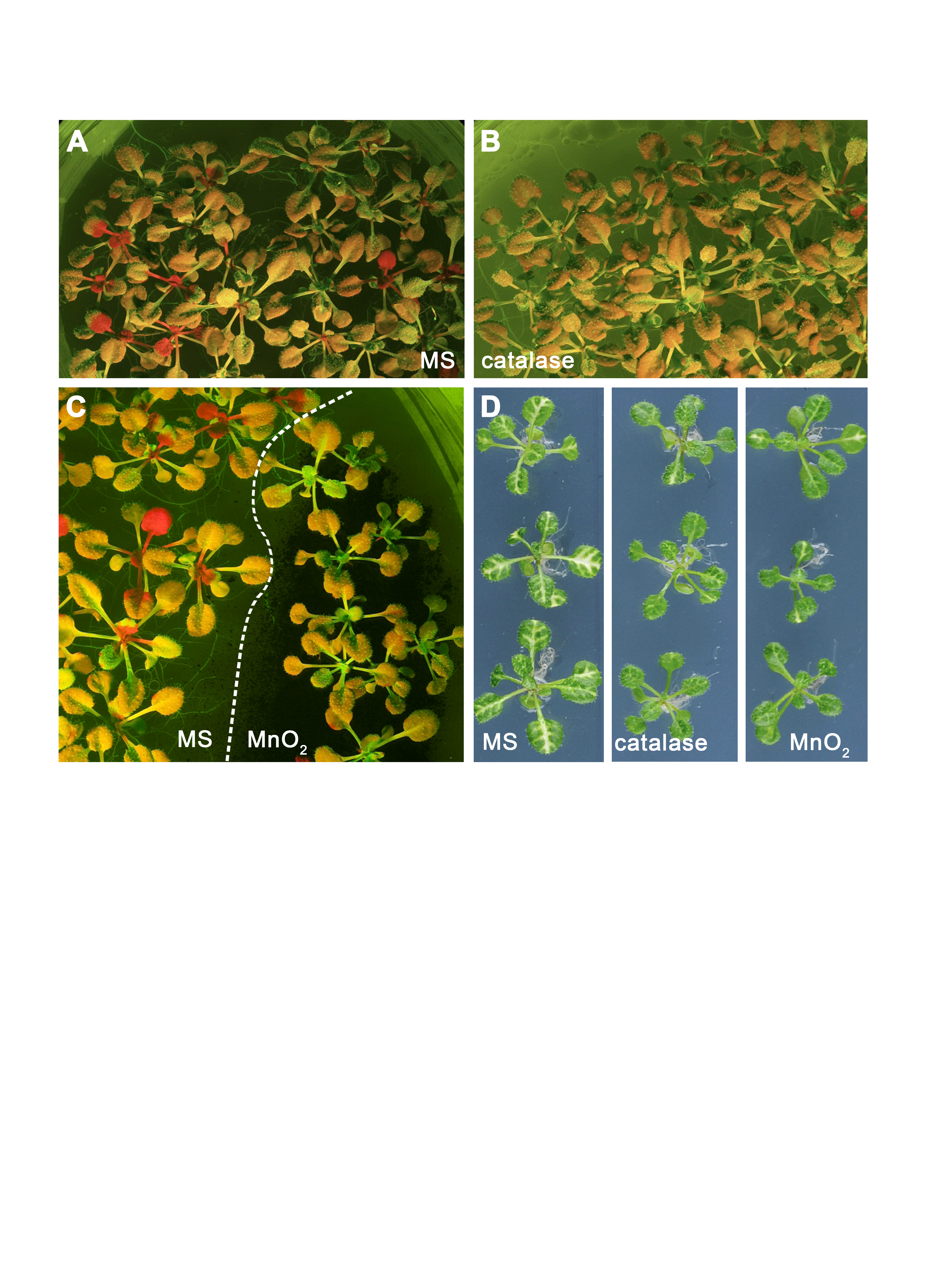

Supplement: Figure S6 — (A) RtSS on Dex-induction medium only. (B) RtSS on Dex-induction medium containing 1 mg/ml catalase. (C) RtSS plants on Dex-induction medium were treated with MnO2 (black powder; right) and the untreated plants to the left of the dashed line were used as controls in the same plates. (D). Three representative 18-day-old AtSuc2:PDS plants grown on MS medium (left), medium containing 1 mg/ml catalase (centre), or medium containing MnO2 (right). (A)–(C) fluorescence images; (D) brightfield images. [file peerj-02-701-s006.png]
