# Supplementary material for: Mobile gene silencing in Arabidopsis is regulated by hydrogen peroxide
Source: PeerJ. 2014 Dec 23;2:e701. doi: 10.7717/peerj.701 (PMC4277490; doi:10.7717/peerj.701)
Supplement: Table S1 [file peerj-02-701-s008.docx]

Liang, White Waterhouse; Mobile silencing is regulated by peroxide

| Table S1. Primers used for genotyping and genomic sequencing | |
| --- | --- |
| primer name | sequence |
| 1g001of1: | 5'CATTGTTCATTGGCACTGACCCTT3' |
| 1g001or1: | 5'CCTGTAATGTATTTGTTAATATGCCTCCTC3' |
| 1g002of1: | 5'AACGGGTTTGTGCTGAACTTGGAT3' |
| 1g002or1: | 5'ACCTATGGACGAGTCTTACGCTAC3' |
| 1g003of1: | 5'TTGTAATAGTATAGCACATCAGTTAGCCTCT3' |
| 1g003or1: | 5'ATGCCAACCAATAGTATCCTCCAT3' |
| 1g004of1: | 5'ACCTAACTTGTCGCATCGGAAAC3' |
| 1g004or1: | 5'CAAATCGGAGAACTTGATACAGC3' |
| 1g005of1: | 5'TGATATTCCAACCGATACTTCC3' |
| 1g005or1: | 5'ATCCAGGCATAAATCCTCTACT3' |
| 1g01of1: | 5’GACGCTGTCGTTTTCACCGTAAGCATCA3’ |
| 1g01or1: | 5’CGAGGTCCTTAAGATCAAGTCCTTGGTTTGC3’ |
| 1g02of1: | 5'ATCGAATGCGTGAAGAAATCGACACTA3' |
| 1g02or1: | 5'CACTCAAGTAAATTAAAGCCACCGACA3' |
| 1g03of1: | 5'CTGATAGGCTACTTGGCTGAGGATGAC3' |
| 1g03or1: | 5'ATGAAGATGATGATGATTTCCACGAGA3' |
| 1g04of1: | 5'ACTTACTTGTCATGTGCGGGAGTCTTGT3' |
| 1g04or1: | 5'ATTTCATCTTCGGAAGTGGAACCTCTAT3' |
| 1g05of1: | 5'TAACGAAGTTGAGAAGGAAACGGATTTAA3' |
| 1g05or1: | 5'GCACGGAGGTGTTGGCTGATAGCTTGTAT3' |
| 1g06of1: | 5'ACGGCGTCAGTGGTGATGATCTGGACAAT3' |
| 1g06or1: | 5'AGCTCATCTAGAAACACCCTCGTTAATAT3' |
| 1g07of1: | 5'CGGGAACGGTTTACTCATGGACTTATA3' |
| 1g07or1: | 5'CATTTCTGAACTCCTCACTTCCGACCT3' |
| 1g08of1: | 5'GAGTTTACAAAGCGGGTGGTGGGAGAA3' |
| 1g08or1: | 5'ACGTGGTTGGAGACGGTATTAGAGTGA3' |
| 1g09of1: | 5'CATATTTTAACCAATCAGAACCCGAGGAT3' |
| 1g09or1: | 5'ACGGTGCCCATGAGATCGGAGTGCCATA3' |
| 1g010of1: | 5'TTGGGTTTAATGAGTACGGGTCTCAGGG3' |
| 1g010or1: | 5'GACTTTGCAGCTCGGGCAGATGTAGGAT3' |
| 1g011of1: | 5'CAAGCATTGTTGGTTGCGTTCCCTTTGT3' |
| 1g011or1: | 5'CGTCACTGCCGTTGTATCTATGGTCCTC3' |
| 1g012of1: | 5'CAACAATGAAACGATTAAATCCTGGCAATA3' |
| 1g012or1: | 5'CTTCAACGGTGACAACATCGGTTACCTC3' |
| 1g013of1: | 5'ACTCTGCTTTCTTGCTCCCATTGCTTCC3' |
| 1g013or1: | 5'TAAACCTCTACATGCCTTCTCGCCTCGA3' |
| 1g014of1: | 5'ACGGTTGACTTTCTGGATACATTCTTGG3' |
| 1g014or1: | 5'GATGCTTACATTCACGCGATTAGACGAG3' |
| 1g015of1: | 5'GTTTGCTTCCTCCCTCTGCTTGTCTTTC3' |
| 1g015or1: | 5'ACTTGACTTACACTGGGCTTCAGGCTCT3' |
| 1g016of1: | 5'CATGATGAGATGAGGCAAGATGTTGTGA3' |
| 1g016or1: | 5'GTGCTATGACCTCTGCGATTTCATTGTT3' |
| 2g01of1: | 5'AGAAACCGAGTCAGTGGTAAGAAAATAG3' |
| 2g01or1: | 5’TGCTATTACTCTCTCCTCTTCATTTCAA3’ |
| 2g02of1: | 5’GAGATGGGTTGAGGTTGAGGATCTTGGG3’ |
| 2g02or1: | 5’TGGAGGCCAAAACAACTTGGCATATTCA3’ |
| 2g003of1: | 5’ATCATCTGCACCCTCAGGGATTTCTC3’ |
| 2g003or1: | 5’TTGCTCGTATTGCTGAATAGCTTCTGACTT3’ |
| 2g04of1: | 5'CTGTAGCTTCTGAGGCAAGAGTAAGG3' |
| 2g04or1: | 5'AAGTAGTATTGGGCACTGGGCGTTT3' |
| 3g01of1: | 5’GCCGGGAACGAACGAAGCTGAAGCTGAA3’ |
| 3g01or1: | 5’AGCTGCTTCCGCGGTGCATCGTCATGTA3’ |
| 3g02of1: | 5'GCAGAGGTGGTAGAAACAGGGATGA3' |
| 3g02or1: | 5'CAAACCCTTACCTGATGTTGTGGAT3' |
| 3g03of1: | 5'GGTTTGGTCCTGGAAGTCGGATTAT3' |
| 3g03or1: | 5'TTGGTAGTGTTCTTTCCCACTCTGG3' |
| 4g01of1: | 5'AACTGAAACAATCTCGAATCATCATCTT3' |
| 4g01or1: | 5'GTTCTGAAAGCACCAAAACCTACAGAAA3' |
| 4g02of1: | 5'GTCTATGCTCATACGCAAACTTGACATT3' |
| 4g02or1: | 5'TTGTAGTCTTTGAGGAGGACACGGAGGC3' |
| 4g03of1: | 5'AGAGCTGAAAGGCAAGAAGCGGGAGAAA3' |
| 4g03or1: | 5'GAGACTTGGCACCTGATGTTACCGACCA3' |
| 4g04of1: | 5'GCTAACCTGGTACATCAAAGAAGTGGTC3' |
| 4g04or1: | 5'TGTCCTGACGCTACAAGTTCTGTGAGTG3' |
| 5g01of1: | 5'AAGTTCTAGGTAGAAGCAGCCATGGAAC3' |
| 5g01or1: | 5'CAGAAAATTTGGCAGGGTGATTAACTTT3' |
| 5g02of1: | 5'TTATCTTCACAGCAAGCCACAGCATCT3' |
| 5g02or1: | 5'GGAGCAAGCAAGGAAACTTATTCTCACTA3' |
| 5g03of1: | 5'GCGTGTAATCATCTCAGGTAAATCCG3' |
| 5g03or1: | 5'TCTACGTTGCTGGAGGTGGTGGTGG3' |
| genomic primers: | |
| podc0f1: | 5'AGACATTCTCAGGCCTTTTATAGATCACCC3' |
| podc0r1: | 5'AAAGCGGCCGCCATACGAAGGAATGATACAAACTC3' |
| podcfusion0f1: | 5'ACATGTACATGTAGGGTTGTGATGGATCAGTGC3' |
| podcfusion0r1: | 5'AAAAAGCTTGGTACCGAATTCCCTAGGACTATTTGCAACGGAACATTGCCTCCTAACC3' |
| art270lacr1: | 5'TGAACGCGCAATAATGGTTTCTGACG3' |
| art270lacf1: | 5'CGTTTGGAACTGACAGAACCGCAAC3' |
| ore10podc0cof2: | 5'TTTAAGCTTTTAGCTTAGATGGACCCACTTCTACTACC3' |
| ore10podc0cor2: | 5'TTTCTCGAGACTATTTGCAACGGAACATTGCCTCCTAACC3' |
| T-DNA insertion line: | |
| lborder0f1: | 5'ATTTATAAGGGATTTTGCCGATTTCGGAAC3' |
| lp0f1: | 5'TTGATTTATGGACCAAACATTCACCATC3' |
| rp0r1: | 5'CTCTACGATGGTCTTGTTATCGTTGAGG3' |
| rp0r2: | 5'AGACGAGCAGTGAGATACACCAATAGTG3' |
| rborder0f1: | 5'AACACGGCGGCATCAGAGCAGCCGATTGTC3' |
